# Ten years of molecular ballistics—a review and a field guide

**DOI:** 10.1007/s00414-021-02523-0

**Published:** 2021-02-16

**Authors:** Jan Euteneuer, Cornelius Courts

**Affiliations:** Institute of Forensic Medicine, University Hospital of Schleswig-Holstein, Arnold-Heller-Strasse 12, 24105 Kiel, Germany

**Keywords:** Molecular ballistics, Backspatter, Ballistic model, Forward spatter

## Abstract

Molecular ballistics combines molecular biological, forensic ballistic, and wound ballistic insights and approaches in the description, collection, objective investigation, and contextualization of the complex patterns of biological evidence that are generated by gunshots at biological targets. Setting out in 2010 with two seminal publications proving the principle that DNA from backspatter collected from inside surfaces of firearms can be retreived and successfully be analyzed, molecular ballistics covered a lot of ground until today. In this review, 10 years later, we begin with a comprehensive description and brief history of the field and lay out its intersections with other forensic disciplines like wound ballistics, forensic molecular biology, blood pattern analysis, and crime scene investigation. In an application guide section, we aim to raise consciousness to backspatter traces and the inside surfaces of firearms as sources of forensic evidence. Covering crime scene practical as well as forensic genetic aspects, we introduce operational requirements and lay out possible procedures, including forensic RNA analysis, when searching for, collecting, analyzing, and contextualizing such trace material. We discuss the intricacies and rationales of ballistic model building, employing different tissue, skin, and bone simulants and the advantages of the “triple-contrast” method in molecular ballistics and give advice on how to stage experimental shootings in molecular ballistic research. Finally, we take a look at future applications and prospects of molecular ballistics.

## What is “molecular ballistics”?

Ballistics, as the science of the motion of projectiles, can be divided into interior ballistics, external ballistics, and terminal ballistics. Interior ballistics studies the projectile while still within the gun; exterior ballistics examines the projectile’s movement through air; terminal ballistics addresses the penetration of and interaction with solids by the missile [1]. Therefore, all ballistic subdisciplines can deliver essential insights for the forensic investigation of gun-related crimes: The shooting of a biological target with a firearm generates a complex and highly informative overall pattern of evidence, comprising impacts, traces, and stains that emerge from the firing of the gun and the interactions of projectile, hit target, and surrounding. These include but are not limited to gunshot residues (GSR), a temporary wound cavity, and a wound track within the target, as well as the so-called forward spatter and backspatter, their relative amounts, composition, extent, distribution patterns, and sites of consolidation.

GSR are mainly composed of burnt and unburnt organic or inorganic particles from the explosive primer from the cartridge, the propellant, and possibly fragments of the bullet, cartridge case, and even the firearm, that will frequently contain Sb, Ba, and Pb or Zn, Cu, and Ti [2]. They can be recovered not only from the hands and clothes but also from, e.g., the nasal mucus of a person who discharged a firearm as well as from the target hit by the shot [3, 4]. However, GSR are primarily physical and chemical traces that are detected, investigated, and quantified using microscopy, chemical analytical, and chemometric methods, e.g., scanning electron microscopy, energy dispersive X-ray spectroscopy, flameless atomic absorption spectroscopy, and energy dispersive X-ray fluorescence [5–7]. GSR analysis may yield information about the type of ammunition, the shooting distance, and the spatial position of the discharged firearm in relation to its surroundings and target.

Wound tracks, meanwhile, i.e., the permanent paths that projectiles leave in traversed tissues, as well as entry and exit wounds are the main subjects of *wound ballistics* which can be considered a subdivision of terminal ballistics concerned with the motions, effects on, and interactions of a projectile with organic tissue [1]. Wound ballistic investigation, e.g., during medico-legal autopsy, of entry and exit wounds, wound tracks, permanent cavities, and the more peripheral extravasation zones (where crushing and laceration by direct contact with the projectile has not occurred), can facilitate the inference of shooting angle and distance, caliber, kinetic energy, shape (and deformation), type, and mode of movement (e.g., tumbling) of the projectile [8].

**Fig. 1:**
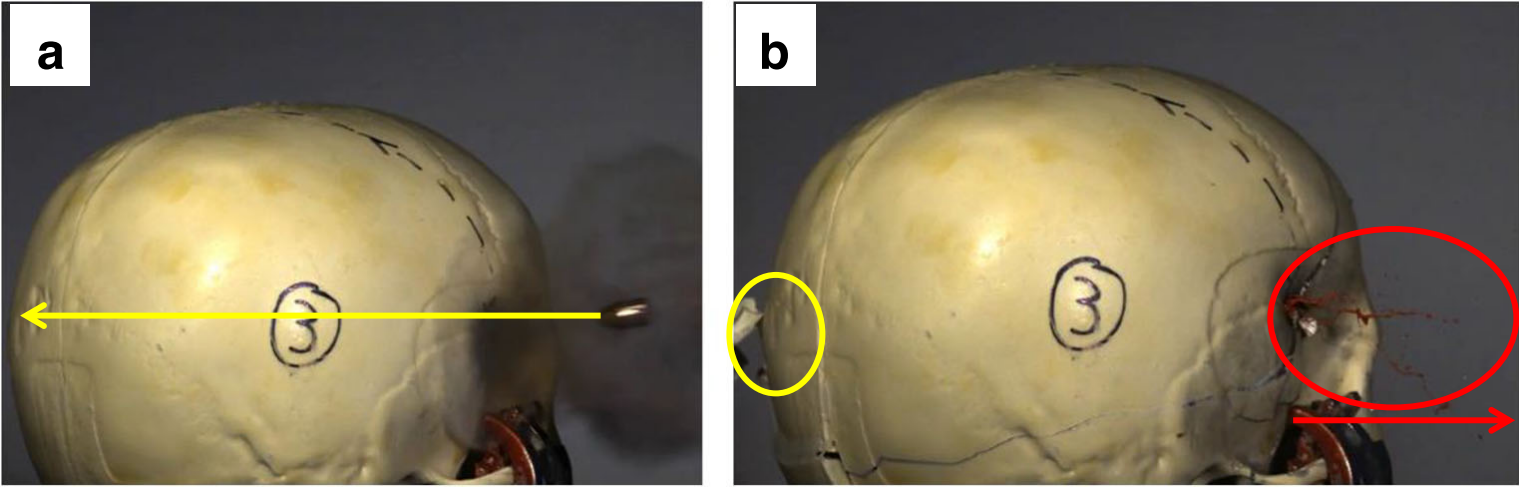
Highspeed image of backspatter generation; **a** the bullet coming from the right side shortly (0.12 ms) before hitting the skull model, **b** 17 ms: the bullet exited the skull model through the exit wound (yellow circle) and backspatter is being propelled out of the entry wound (red circle) contrary to the bullet path (red arrow)

**Fig. 2:**
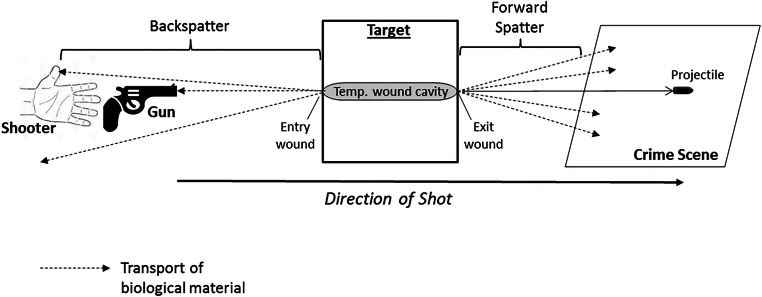
Schematic depiction of the possible distribution of forward spatter and backspatter caused by a shot at a biological target

*Molecular ballistics*, in contrast, is defined as applying the methods and techniques of forensic molecular biology to the analysis of biological traces that are generated when the interaction of firearm projectiles with hit targets produces “forward spatter” and/or “backspatter.” Such has been termed the biological material that is propelled out of the exit and entrance wound, respectively, of a biological target hit by a firearm projectile. While the emergence out of the exit wound of forward spatter accompanying the projectile is straightforward to comprehend, the formation of backspatter is less intuitive: it is generated due to the combined forces of several interacting wound ballistic effects including (1) the elastic collapse of the temporal wound cavity and the concomitant equilibration of resulting overpressure [9–12], (2) a stream of liquid and tissue particles accelerated along the lateral surfaces of the projectile into the direction of the entry wound, called “tail splashing” [9, 13, 14], and (3) for contact shots, the ejection of muzzle gases out of the entry wound from the powder cavity [9, 15, 16]. Hence, forward spatter and/or backspatter are cast along and contrary to the bullet’s trajectory, “forward” into the direction of flight of the projectile and “back” to the shooter and the weapon, respectively (Figs. 1 and 2).

The term “molecular ballistics” was coined when about 10 years ago Courts and Schyma started investigating whether backspatter can be found and recovered from inside surfaces of firearms that had been used to shoot at biological targets. The phenomenon of backspatter had been discovered and described by Weimann as early as 1931 [14], but Courts and Schyma were first to show that traces of backspatter do consolidate on and can be recovered from inner surfaces of firearms after contact shots and reliably serve as a source of DNA eligible for forensic analysis from both ballistic models [17] and in real cases [18]. Applying a method inferred from their findings in an investigation of a case of multiple familial homicides, they demonstrated the method’s potential and emphasized the necessity to routinely include sampling *inner surfaces of firearms* for traces of backspatter in routine forensic casework involving gunshot-related crimes [19].

In the following years, molecular ballistic research had its scope expanded considerably, and it was demonstrated that not only nuclear DNA but also mitochondrial DNA (mtDNA), messenger-RNA (mRNA), and micro-RNA (miRNA) can simultaneously be recovered and analyzed in parallel from traces of backspatter [20]. Also, it was established by Grabmüller et al. that backspatter could be retrieved from within firearms and successfully be analyzed even if the shooting distance had been considerably larger than encountered in contact shots or near contact shots, i.e., up to 30 cm [21]. Moreover, by integrating forensic RNA analysis-based organ tissue identification (OTI) [22] into molecular ballistics, Lux et al. could show that it is possible to identify shots to the head by detecting brain-specifically expressed miRNA in traces of backspatter [23]. This was later expanded by Sauer et al. to infer shots to other hit locations, e.g., the torso by detecting miRNA specifically expressed in heart or lung tissue in backspatter [24]. Parallel to these endeavors, topological and cumulative methods for the simultaneous recovery of backspatter and GSR from shooters’ hands were compared [25, 26] to help optimizing evidence collection for molecular ballistic analysis.

**Fig. 3:**
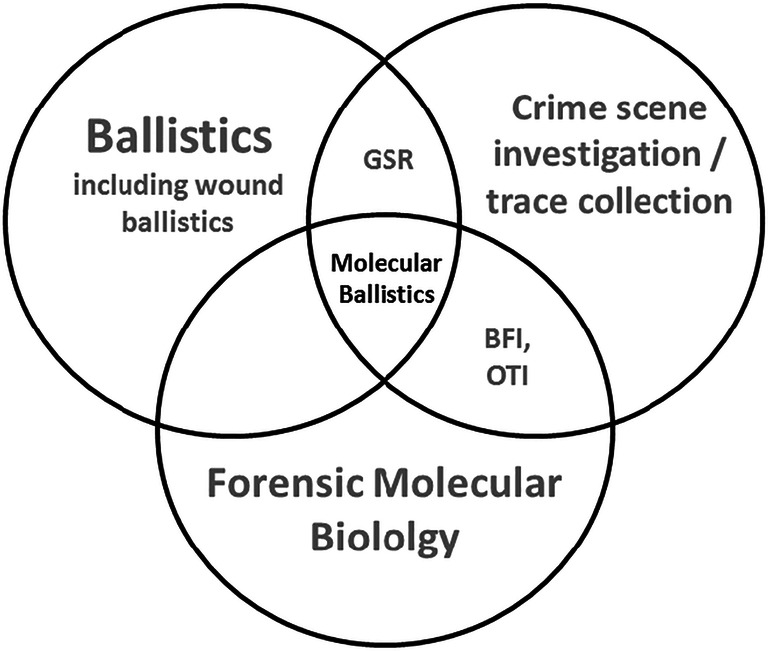
Molecular ballistics as transdisciplinary intersectional approach

Recently now, Gosch et al. analyzed DNA traces recovered from firearms wielded in realistic, casework-relevant handling scenarios to improve the understanding of factors affecting the variability of trace DNA characteristics recovered from firearms handled in gun-related crimes [27]. By showing that trace DNA characteristics differed distinctly between handling conditions, firearm and surface types, as well as handling individuals and intra-individual deposits, they provided useful insights for forensic experts evaluating alternative activity-level propositions in gun-related crimes and hence connected molecular ballistics with routine forensic DNA analysis. Taken together, molecular ballistics can be perceived as a transdisciplinary approach operating at the intersection of terminal ballistics, crime scene investigation, and forensic molecular biology (Fig. 3).

The aim of applying molecular ballistics is to fully and in-depth exploit all available biological evidence from scenes of gun-related crimes and to provide a multi-dimensional molecular analysis to support the *individualization and contextualization* of traces and trace patterns generated by shots at biological targets which then together with wound ballistic and GSR analyses may critically contribute to an evidence-based, objective reconstruction of the course of events and inference of involved persons.

## An application field guide

### Where to look

The scope of molecular ballistics encompasses all traces of biological material ejected from a body by wound ballistic effects that is hit by a high velocity projectile. In general, the pattern of blood and tissue traces at a scene of gunshot injury is dependent mainly on whence they left the body: from the entry site or, if present, the exit site of the bullet. In technical terms, projectiles that only enter but do not exit the body create “penetrating wounds,” while projectiles that fully traverse and then leave the body or one of its parts create “perforating wounds” [28], yet the former is commonly applied to all gunshot trauma. Apart from that, wounds caused by grazing shots may be a source of biological trace material as well and molecular ballistic analysis can help to distinguish traces of damaged tissue from other wounds, e.g., in events with multiple shots and injuries.

Biological material found at a crime scene involving gunshot injury can be analyzed to help reconstruct contextual details of the course of events of the crime—for example, to infer the number of persons involved or whether the victim was moving or being moved after being hit by the shot. Still, considering each gunshot isolatedly (disregarding complex situations like a projectile perforating an arm and then re-entering the body at another site), there are two directions into which biological material can be propelled, as laid out before: following the projectile through the exit wound in the direction of flight (forward spatter) and from the entry wound in the direction opposite to the trajectory (backspatter). Both exiting and entering projectiles are capable of causing the expulsion and distribution of biological material in comparable angles, yet with different amounts and spatter velocities [29] causing different stain patterns. The morphological, spatio-relational analysis of such stain patterns is the subject of “blood pattern analysis” (BPA) which is not touched upon this review and for which extensive literature is available ([30] provides a good overview, for a description of different subcategories of BPA, see [31]). However, while the interpretation of blood stain patterns is difficult, prone to error and cognitive bias, and hence needs to be performed with great caution (e.g., [32–34], also see, [35] for a comprehensive list of references), the main focus of molecular ballistics lies on the molecular biological analysis of the traces which are in fact there “as is,” while abstaining from hypothesizing how exactly they got there.

#### Backspatter

Depending on the shooting distance, backspatter traces are often found on the shooter’s clothes and hands in particular, whence they can be collected. Yet, as pointed out, the center of molecular ballistics’ interest is the firearm used in gunshot-related crimes and incidents. This central object often connects the victim(s) and the perpetrator, therefore traces recovered from there may be of substantial evidential weight. In fact, backspatter traces can consolidate on basically all outer and inner surfaces of a firearm and when manifested as microspatter (droplet size < 0.5 mm diameter [36]) may even be invisible for the unaided eye but still yield DNA profiles [18, 19, 37, 38]. Thus, when collecting evidence for crime reconstruction, all surfaces of a firearm should be carefully investigated, and all detachable parts should be disassembled and evaluated for traces as well.

Especially, but not only after contact shots, is it highly advisable to investigate the inside of the barrel for backspatter traces. This inner surface is well protected from environmental contamination and from direct contact with, e.g., packaging material which could lead to loss of biological material [39]. In a systematic study in 1992, Stone investigated 1200 cases of suicides involving firearms and detected blood inside the barrels of 53% and 57% of the involved revolvers and pistols, respectively. He also performed test firings and could detect blood inside the barrels of 40% and 42% of the respective firearms even after firing one shot [40]. The percentages of blood traces detected on the outside of the barrel were even higher, ranging from 74% (revolvers) to 76% (pistols). However, Stone used Leucomolachite Green to detect blood which is less sensitive than current PCR-based DNA quantification methods. The latter was applied by Schyma et al. in the investigation of 20 cases of suicide by gunshot of which 17 yielded successful DNA profiles after sampling the insides of the barrels. Second shots were performed as well and in 14 cases, DNA profiles of sufficient quality were obtained [18]. Prior to sampling, the barrels in some cases had been examined endoscopically to assess the distribution of the traces within the barrel. As the barrel contains residues of the combustion process like soot or metallic particles, which are known to inhibit PCR, it can be beneficial to focus trace collection exactly to the spot where the biological material is sitting and thus minimize the concentration of possible inhibitors on the swab. Furthermore, the efficiency of the sample collection method can be evaluated by endoscopic control. Schyma recommends the investigation of the barrel by an endoscope with 0° angle, e.g., the Hawkeye Borescope (Gradient Lens Corporation, USA) with a viewing angle up to 42°, which allows a straight view through the barrel. A mirror tube can be applied as well to enable a 90° orthogonal view while keeping the diameter below 5 mm, so that even small-caliber weapons can be assessed. Yet for proper documentation, a stronger light source and additional camera equipment is required, e.g., the Endolight FOT Xenon (Eltrotec, Germany) and a Leica MC170 HD (Leica, Germany). The latter needs additional adapters to connect to the endoscope but creates the best image and video quality via HDMI connection to a suitable monitor. Video documentation in HD is preferred for estimating the distances and stain sizes inside the barrel, and taking screenshots from the video is also possible, replacing single photos [41].

#### Forward spatter

As forward spatter is trailing the projectile out of the exit wound from perforating shots, unlike backspatter, it cannot consolidate on the shooter or the fired gun. Therefore, forward spatter can only be found on the crime scene (including objects and bystanders) and is a main subject for BPA in investigation of gunshot-related crimes. Consequently, documenting and securing traces of forward spatter is a task for crime scene investigation personnel who should be (made) aware of the significance of these traces. While no molecular ballistic research focusing solely on forward spatter has been published so far, all information gathered from the molecular biological analysis of such traces can be compared and complemented with information from traces collected from absent and later secured guns, perpetrators, or perhaps even victims. Also, in cases where the circumstances of a crime are unclear or evidence or witness reports are contradictory, molecular ballistics employing forensic RNA analysis (see “Analysis of RNA”) can support contextualized event reconstruction by the molecular differentiation between tissue origins in traces of forward spatter where BPA has been the only option so far, e.g., to help the discrimination between blunt impact or gunshots [42].

### Distribution and distance of backspatter

There are two main questions of criminalistic relevance connecting shooting distance and backspatter. (1) Up to what distance can traces of backspatter be recovered from the firearm and/or the shooter and what variables (weapon type, caliber, shooting angle, etc.) may influence this? (2) Is there a correlation between shooting distance and (any aspect of) backspatter that can be used to infer the former from the latter?

To answer these questions, several studies have been conducted so far. For instance, Karger et al. performed experiments using a 9-mm SIG P210 pistol and two kinds of 9-mm Luger ammunition to shoot at the heads of calves from distances of 0 to 10 cm and found instances of macrobackspatter (droplet size > 0.5 mm diameter) and microbackspatter (droplet size < 0.5 mm diameter) up to distances of 119 cm and 69 cm, respectively [36, 43]. In a series of shots to the occipital bone of anatomically correct skull models doped with a mix of blood and contrast agent soaked into a spongious matrix, Euteneuer et al. detected backspatter traces in various amounts on the inner and outer surfaces of the handguns fired from distances increasing from 0 to 50 cm, containing sufficient trace material for successful DNA profiling in 81% (Glock 19, 9 mm Luger) and 76% (Smith&Wesson CTG, .38 Special) of the shots, respectively, while observing backspatter traces on the floor in several-meter distances from the skull model [38]. A qualitative study by Grabmüller et al. described the successful analysis of backspatter traces recovered from the inner surfaces of the weapons (a revolver (Smith&Wesson, .38 Special) and two pistol models (Astra 9 mm and FEG 7.65 mm Browning both loaded with 9 mm Luger) after shots from up to 30 cm distance at polyethylene bottle models filled with gelatin and doped with a mix of blood and brain tissue [21]. Obviously, reality is represented more closely by case reports involving real human bodies. However, while many reports of cases of suicide by gunshot are available with a description of backspatter traces found on the gun, hand, or proximity of the victim, rarely could the shot distance be inferred reliably. Still, one case report described a suicidal shot to the head (Sig-Sauer P6, 9 mm pistol firing ammunition Quick Defense “Polizei-Einsatz-Patrone”) that produced extensive backspatter up to a distance of 4.6 m from the body comprising blood and brain tissue, as well as backspatter on the ceiling of 2.5 m height [44]. At a rare occasion, Rossi et al. had the opportunity to shoot at the head, reinfused with bovine blood, of a deceased male who had donated his body to research to study backspatter pattern production. The amount of backspatter on a board, which had been placed about 45 cm away from the head produced by a shot with a .45 1911 style pistol (Les Bear Custom, Model Concept 4) was considerable, confirming experimentally the creation of backspatter in amounts and travelling distances observed in case reports [45]. Taken together, these studies show that backspatter will reach the shooter and/or gun in most typical shooting incidents from typical distances; however, the probability with which it consolidates on surfaces where it can be found cannot reliably be calculated at the current state of knowledge and data.

### Sample collection

As firearms are—with very few exceptions—made of metal or alloys with antirust or anticorrosive coatings like browning gas nitriding, teniferation (ferritic nitrocarburizing), etc., all sampling techniques are applicable that are recommended for non-porous/non-absorbent materials. To this date, a comprehensive study to establish the optimal sampling technique, comparing swab brands and material as well as extraction methods for different biological materials (blood, different tissues, bone splinters) potentially comprised in backspatter traces has not been performed. In a rather superficial study, Wood et al. still found that the recovery from acellular human DNA with both cotton and nylon swabs from “firearm metal” (sic) was inefficient, only at about 15% of the applied amount of DNA [46]. Therefore, the sample collection technique should be optimized. Hedman et al. showed that when using the double-swab technique [47], the first wet swabs yield 31-fold and 28-fold higher DNA concentrations when collecting dried saliva from brass and steel, respectively [48]. In two unrelated comparative studies, Bruijns et al. [49] as well as Comte et al. [50] demonstrated that nylon-flocked swabs like the 4N6FLOQ Swabs Genetics (Copan, Italy) exhibited a better recovery efficiency with saliva and touch DNA than other swabs. It is thus advisable to employ a double-swab technique or at least use a moistened swab combined with flocked nylon swabs for trace collection. However, further systematic in-depth research would be desirable. Although most molecular ballistic studies were conducted employing forensic cotton swabs instead of nylon swabs moistened with sterile, desalted water [17–21, 23, 51, 52], they produced acceptable results. In a recent study, Schyma et al. used first a dry swab for sampling backspatter from inside the barrel directly after the test shots while the backspatter traces were still moist, followed by a second wet swab [53]. They also highlighted the difficulty in proper nuclease-free weapon cleaning, especially for RNA residues. Alternatively, a modified version of the double-swab technique has also been applied frequently, with the head of one single swab moistened on one half while leaving the other half dry [20, 21, 37, 38]. This approach increases the sample concentration on the swab but should be adapted to the amount of material found at the respective weapon. Also, pooling of sampled material by combining several swabs in one lysis volume can, if applicable in a given case, improve DNA yield in situations with minimal trace amounts [18].

At this point, it needs to be emphasized that the recovery of backspatter traces for molecular ballistic analyses should *always be performed in parallel* to sample collection for standard forensic DNA analysis, e.g., from the firearm’s grip, trigger, etc. to facilitate individualization which is essential for inferring the weapon’s handler. How “touch DNA” (e.g., from epithelial cells) is transferred to a gun by its handler(s) via touch/direct contact in different realistic mock case scenarios and how to collect it has recently been investigated in-depth by Gosch et al. [27] as mentioned above.

**Fig. 4:**
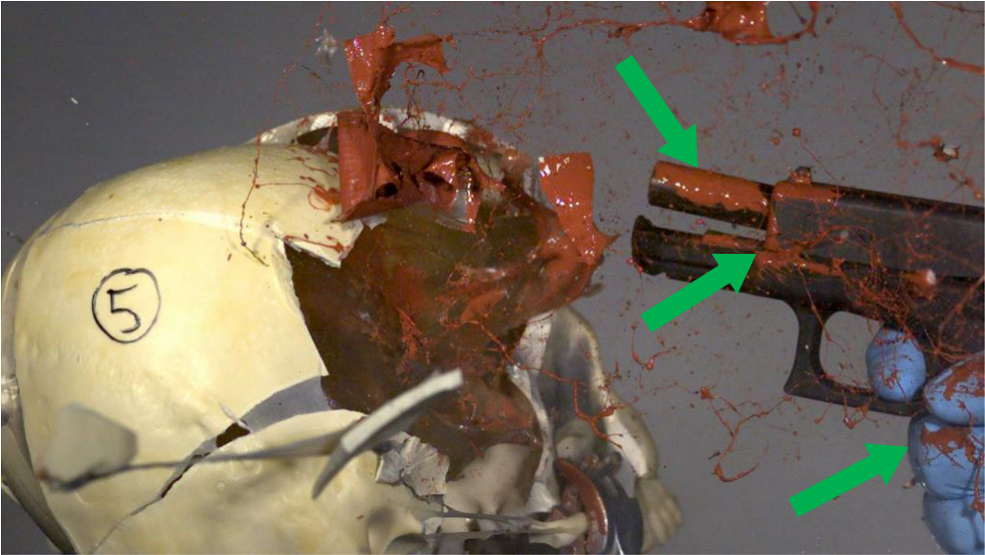
Modified from [37]. Twenty milliseconds after contact shot with a Glock19 and 9 mm Luger ammunition to a skull model with gelatine brain simulant and doped with a plastic bag filled with “triple-contrast” mix. Arrows indicate the splashes of contrast mixture on the outer surface of the barrel and small gaps while the slide is still pulled back by the shot and on the hand of the shooter

The search for and collection of traces should be performed “from the outside to the inside,” i.e., first, all outer surfaced should be carefully investigated and sampled, even where no traces are visible (minding “touch DNA” and microspatter). Afterwards, the weapon should be disassembled (if possible) and samples should be retrieved from detachable weapon parts, especially from “inner” surfaces (i.e., that are not exposed to the outside of the gun when fully assembled), which have proven to bear DNA-containing material that allows for successful victim identification [19]. In pistols, the outer surface of the barrel, which is covered and protected by the slide, is only shortly exposed during the shooting process. This short interval of exposition was shown to temporally correlate with the backspatter trajectory so the outside of a pistol’s barrel may “catch” backspatter particles which then consolidate and remain there covered by the slide until collected [37, 38] (Fig. 4). Also, the barrel outside and other inner surfaces except the barrel inside are less exposed to and affected by the physical and chemical stress caused by secondary and further shots with the same weapon that may destroy or remove trace material that had consolidated on the inner surface of the barrel after the first shot [19].

The inside of the barrel, on the other hand, is the surface not only best protected from the outside but also the most difficult to sample, as pressure required for efficient sample collection can hardly be applied onto the swab. Additional methods should thus be considered in cases where traces are detected (e.g., with the help of an endoscope) but cannot properly be recovered using swabs which will frequently be the case with long barreled firearms. For instance, a patented tool devised and specially dedicated for the efficient and quantitative collection of backspatter traces from inside gun barrels is called “GunSwab C1” which is available for handguns and long barreled guns of different calibers (Coloprint, Germany). The device consists of DNA-free felt pieces attached to a metal pulling rope that can be pulled through the entire length of the barrel, thereby applying pressure and brushing loose and collecting traces and residues sticking to the barrel surface.

### What can and should be investigated

#### Analysis of DNA

In simple terms, in molecular ballistics, DNA-based individualization of biological material should help to answer the question “Who shot whom?” and short tandem repeat (STR) profiling from backspatter traces has already been shown to enable identification of victims of gun violence, even from complex mixed traces [19]. DNA profiling from such traces, whether found inside the weapon or on the suspect, is of even higher importance in cases where the victim(s) is/are absent or has/have been removed from the crime site or when the firearm had been thrown away and later found at a place unrelated to the crime site or in cases where the body/bodies has/have been rendered visually unidentifiable by putrefaction, mutilation, burning, etc. If the victim is unknown and his/her DNA profile is not represented in any accessible database, forensic DNA phenotyping (FDP) can additionally be performed to aid investigations by providing clues to externally visible characteristics (EVC) of the deceased, like eye color, hair color, and skin color [54] and by determining his/her biogeographical ancestry (BGA) [55]. Notably, both, determination of EVC and BGA can also be performed for touch DNA samples recovered from the gun [27] to provide investigative leads in the search for an as-yet unknown suspect.

Different methods have been employed to extract DNA from backspatter samples collected from firearms, but given that neither has a comparative study of different methods as yet been performed, nor is exact replication of the backspatter generation even in replicate shots under identical conditions possible [38], it cannot be stated which method is best suited. As laid out before, PCR inhibitors are always present in gunshot residues, therefore kits including stringent washing steps to remove inhibitors are recommended, e.g., the PrepFiler DNA Extraction Kit (ThermoFisher) that has repeatedly been used for molecular ballistics [17–21, 37, 38, 51, 52]. For quantitative PCR (qPCR)-based DNA quantification and inhibition monitoring, different commercially available kits (Quantifiler Human DNA Quantification Kit (ThermoFisher) [17–19, 23, 51, 52], Plexor HY System [20, 21], and PowerQuant System (both Promega) [37, 38]) have been used, and in no case was strong systematic inhibition reported. Of the mentioned kits, the PowerQuant System is also capable to detect DNA degradation; also, no reports on degradation influencing the outcome were presented. Still, in the case report with a multiple homicide, DNA in two samples with uninhibited PCR may have been largely degraded as no results could be obtained [19]. Hence, degradation may indeed occur and can be expected to result especially from conditions present inside a gun, like spikes of high heat and chemical stress after several additional shots. In these cases, STR profiling with kits specifically dedicated for forensic trace material comprising robust buffers and short STR systems is advisable. For minuscule samples with low DNA yields where STR typing has failed, the additional analysis of mitochondrial DNA was also demonstrated to be feasible and may provide an additional investigative tool [20].

#### Analysis of RNA

**Fig. 5:**
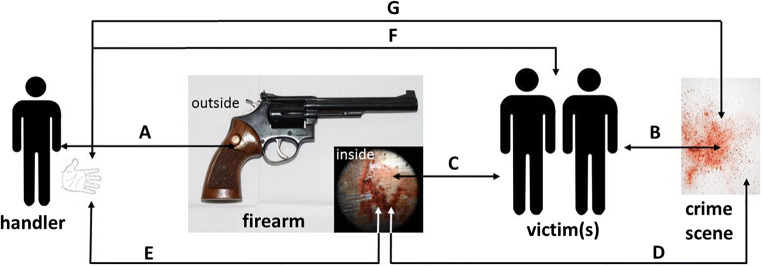
Schematic representation of possible reciprocal relationships in a molecular ballistic analysis. E, (Touch) DNA from direct contact of the shooter/handler with the weapon. B, DNA of the victim matches DNA from forward spatter at the crime site; RNA may provide additional contextual information (e.g., hit location). C, DNA of the victim matches DNA from backspatter inside the weapon; RNA may provide additional contextual information (e.g., hit location). D, DNA from backspatter inside the weapon matches DNA from forward spatter; RNA may provide additional contextual information (e.g., hit location); this connection can be useful if no body/victim is encountered at the crime scene. E, DNA from backspatter inside the weapon matches DNA from backspatter on shooter (hand, clothes); if primary contact with the weapon is disputed, e.g., due to alleged secondary DNA transfer, (E) can establish the connection. F, DNA from backspatter on shooter (hand, clothes) matches the victim’s DNA; RNA may provide additional contextual information (e.g., hit location). G, DNA from backspatter on shooter (hand, clothes) matches DNA from forward spatter at crime site

The origin of forensic RNA analysis may be traced back to 1994 when Phang et al. were first to use RT-PCR to analyze mRNA from postmortem tissues in a forensic setting [56]. Since then, the interest in highly versatile RNA analysis surged within the forensic and medicolegal community and since 2009 also involves miRNA [57, 58]. To the current date, forensic RNA analysis has been creatively applied to an array of different forensic research questions most prominently body fluid identification (BFI) [22] and OTI [24, 59], which as mentioned above is also relevant to and applicable in molecular ballistics [23, 24]. Hence, molecular ballistic investigations when applied to traces of forward and backspatter recovered from, e.g., the crime scene, inside surfaces of the firearm, the hands and/or clothing of the shooter, and when integrating DNA and RNA analyses, including analysis of DNA transfer and touch DNA [27] can populate a dense network of context mediating reciprocal relations and inferences (Fig. 5).

Currently, however, and in contrast to DNA analysis, there is no validated commercially available kit for forensic RNA extraction, and as shown by Grabmüller et al. [60] and Schweighardt et al. [61], the best suited method should be selected beforehand, dependent on the given conditions. Consequently, previous studies described the successful application of various different methods for (DNA/)RNA (co-)extraction in a molecular ballistic context: mirVana miRNA Isolation Kit (Thermo Fisher Scientific) + QIAquick PCR purification kit (Qiagen, Germany) [23], NucleoSpin miRNA Kit (Macherey-Nagel, Germany) + PrepFiler Forensic DNA Extraction Kit (Thermo Fisher Scientific) [21, 25, 26], and PrepFiler Forensic DNA Extraction Kit only [20].

In general, if RNA analysis is to be performed, samples that cannot immediately be processed after collection should be immersed in an RNA-stabilizing reagent (e.g., “RNA later,” Thermo Fisher Scientific) and/or stored at −80°C. If such facilities are unavailable, samples should be stored at no more than room temperature, kept dry and in the dark, and be processed as soon as possible. RNA and DNA can then be co-extracted from the same sample without having to choose DNA over RNA or vice versa [62, 63]. If DNA analysis has to be prioritized, sufficient RNA may still be present in the remaining eluates or flowthroughs from DNA extraction procedures of samples after DNA analysis has been finished [64, 65].

## Research guide

### Safety first—a cautionary advice

Be aware, that *always* when experimental shootings at (molecular) ballistic models are performed, backspattered material and/or splinters or fragments from the bone (simulant)s or even slowed down bullets ricocheting back from the bullet trap may potentially hit and injure the shooter. We strongly advise to adhere to all applicable safety regulations and always have the shooter wear appropriate protective gear all the time.

#### How to make a model

The often-cited phrase attributed to the British statistician George Box, “All models are wrong, but some are useful” is extraordinarily fitting in the context of establishing models for molecular ballistic research or ballistic research involving biological targets in general. The requirements for a realistic model in this field are quite high, as the human body is a complex, heterogeneous structure with considerable inter-individual variation. Persons having their body donated to ballistic research after their death are exceedingly rare. Other biological targets like (dead) animals, animal parts, or animal tissues have been employed in studies but are difficult to replicate due to biological variation and will at best only be a mediocre simulation of the human body and its parts. As an alternative, artificial materials as simulants for biological tissues and structures are available and still being developed that exhibit physical and mechanical properties comparable to the biologic original, and while a perfect simulation of (parts of) the human body in all its complexity and detail including the vascular system appears to be out of reach, those simulants can still be used to compose a model suitable for ballistic tests investigating specific aspects. Already in 2004, Jusilla compiled a list of qualities needed for tissue simulants [66], which is still current and which we reproduce here:

- similarity in the deceleration of the projectile between the simulant and the living tissue the simulant has been validated for
- similarity in the deformation behavior of the projectile
- similarity in the kinetic energy dissipation
- kinetic energy dissipation measurability with reasonable accuracy
- extrapolation of temporary cavity diameter
- elastic behavior similar to living tissue for observation and measurement of temporary cavity formation and tissue compression
- extrapolation of permanent cavity diameter
- reproducibility.

All those requirements should be fulfilled by the models employed in molecular ballistics research; however, further aspects must necessarily be included:

- similarity in the generation of forward- and backspatter (which, in fact, is interconnected with many of the other qualities)
- a source of biological material (tissue/blood), which must in some way be integrated into the model system.

To instantiate all qualities in a single model setup requires an extensive developmental effort, time, costs, and thorough validation with, if possible, several types of firearms. Research facilities with less resources may instead choose to use more simplistic models, implicating only the most important aspects which are needed to simulate the part of the body the planned ballistic test is aiming for. Essentially, these are the primarily involved tissue (muscle or organ), the skeletal part if head, chest, or limbs are considered (represented as a bone structure), and the surrounding skin.

Earlier, less sophisticated models used for molecular ballistic experiments usually consisted of mere gelatin blocks, sponges and acrylic spheres [17], or gelatin-filled polyethylene bottles of different sizes covered with silicon [20, 21, 24]. In recent studies, two model systems were in use: Euteneuer et al. established a head model system employing an anatomically correct polyurethane skull with rubber coating to simulate the periosteum (SYNBONE®, Switzerland) and “triple-and double-contrast” mixes (see below) soaked into a spongious matrix inside an evacuated plastic bag as source for biological material glued from the inside to the skullcap, with ballistic gelatin as brain simulant [37, 38]. This model allows for the most realistic head shot simulations including biological material so far. The preparation, however, is elaborate and time consuming, and the model system lacked an additional skin layer. Schyma et al. devised the “reference cube” model, a ballistic gelatin cube doped with liquid “triple-contrast” mix and covered with a synthetic, absorbent cloth (60% viscose, 20% polyester, 20% polypropylene) [52], which was employed in several studies on backspatter research and gelatin/wound channel behavior [53, 67–69]. Originally intended to be used as a head simulation, the “reference cube,” while resembling the volume of the cranial cavity, is lacking a rigid casing to simulate the skull bone and thus exhibits forward and backspatter behavior that is different from the human head but has the advantages of easy preparation, translucency to facilitate wound cavity observation via high-speed video, and also being suitable to be used as a torso simulation.

Other model systems for ballistic experimentation could be adapted for use in molecular ballistics by adding a source of biological material: For example, Riva et al. presented individual synthetic head models based on real cases of gunshots to the head by using polyurethane plates, ballistic soap, and gelatin with proportions reflecting the respective victims’ tissues as measured by postmortem CCT [70]. Thali et al. developed a spherical “skin-skull-brain” model comprising a silicon cap containing synthetic leather as skin simulant, a polyurethane sphere to simulate skull bone, and latex and ordnance gelatin as simulants for the periosteum and for brain tissue, respectively [71]. A quite comprehensive and anatomically correct skull model including complete skin simulant was set up and thoroughly evaluated by Mahoney et al., but its components were custom made using UK military resources and thus are not easily reproducible and will be out of reach for most academic laboratories [72].

An overview of simulant materials that are or were used or investigated in the context of (molecular) ballistics is given in Table 1. It neither is nor is meant to be comprehensive concerning all simulant materials devised so far in total, as those developed in the context of pure medical or mechanical use are neglected, as well as those materials which were proven as clearly unsuited in comparative studies.

**Table 1:** Overview of different ballistic simulants

Simulant for	Substance (recipe)	Described in^a^	Comment
**Soft tissue**	10% ballistic gelatin	Fackler [73]	Widely used, elastic muscle simulant, also used as brain simulant, e.g., [37, 38, 71]
	20% ballistic gelatin	Misc., no “official” recipe	“NATO formula,” often used in military studies, comparable to 10% [74]
	Ballistic soap	Different recipes, e.g., in [8]	Wound channel research, inelastic behavior, not recommended for backspatter generation
**Brain tissue**	Sylgard gel (Dow, USA)	Zhang et al. [75, 76]	2 component silicone gel, good mechanical properties. Not used in backspatter research so far
	Glycerol/water/starch/fiber	Lazarjan et al. [77]	More closely comparable to bovine brain tissue than gelatin. Not used backspatter research so far
	Agar/glycerol/water	Falland-Cheung et al. [78, 79]	Comparable to deer brain, yet no improvement to gelatin. Not used in backspatter research so far
**Bone**	SYNBONE	Euteneuer et al. [37]	Similar mechanical properties and fracture behavior as real bone; different forms as well as anatomically correct models available
	Sawbone	Bir et al. [80]	Less suitable compared to SYNBONE and human femur
	Acrylic	Courts et al. [17]	Half sphere used as head model. Easy to use and stable, but simplistic with different mechanical properties
	Layered polyurethane	Thali et al. [71]	Distinctly constructed layered sphere with Tabula interna, externa, and diploe. Easy handling due to spheric form, difficult to replicate
	Polyethylene	Grabmüller et al. [20, 21], Schyma et al. [51]	Polyethylene bottles, easy to obtain and handle, stable, but different mechanical properties
**Skin**	Chamois leather	Euteneuer et al. [81]	Used and recommended by the German Federal Criminal Police Office
	Semi-finished chrome tanned upholstery “crust” cowhide	Jusilla et al. [82]	Best skin simulant in a comparison study with 13 materials. Still partly natural product and thus prone to variation
	Silicon with artificial fibers	Thali et al. [71]	Results comparable to real cases
	Dental silicon	Falland-Cheung et al. [83]	Suitable material with mechanical properties comparable to fresh porcine skin
	Lorica leather	Das et al. [84]	Better skin simulant for backspatter compared to natural rubber, at shots with 9 mm Luger
	Roebuck 1518 synthetic chamois	Pullen et al. [85]	Suitable skin simulant for tests with non-deforming bullets.
	Silicon	Misc.	Different kinds of several variations of silicon were often used to cover a model. Cheap, but only for stability use, less for simulating skin

^a^Simulants get improved over time. The here mentioned publications are not necessarily relating to the first mention of the simulant but rather those with biggest implication for (molecular) ballistic research. See main text for more information

##### Tissue simulants—gelatin and alternative substances

All studies on molecular ballistics that are currently available used 10% ballistic gelatin as tissue simulant [17–21, 37, 38, 51–53]. This substance is generally accepted and widely employed as a soft tissue simulant with 10% being the most frequently used concentration for ballistic studies and thus lends itself well for comparison. Furthermore, it is easy to produce, e.g., following Fackler’s instructions of 1988 [73], exhibits elasticity and hence can generate backspatter by facilitating the manifestation and elastic collapse of a temporal cavity (in contrast to, e.g., ballistic soap). Also, due to its translucency, it allows for high-speed video capture and post-shot wound channel evaluation. Some researchers and especially military instead of Fackler’s applied the “NATO formula” with 20% gelatin (e.g., [74], although no citable or official recipe is available in literature) or other recipes; however, this issue has been addressed in detail elsewhere (e.g., [66]) and is not within the scope of this review. As human brain tissue exhibits mechanical properties different from typical soft tissue and gelatin, and with the head being an important object for ballistic research, other simulants for brain tissue have been devised and used, e.g., Slygard gel [75, 76], but have not been employed in backspatter studies so far. Lazarjan et al. presented a mix of glycerol, water, starch, and fiber which exhibit qualities more closely comparable to bovine brain than 3%, 5%, or 10% gelatin, yet they conceded that the formula still needs optimization and that the substance’s opacity is problematic [77]. Falland-Cheung et al. evaluated a mix of agar, glycerol, and water as translucent brain simulant and found it suitable and comparing to deer brain tissue after impact and ballistic test shootings [78], yet demonstrating no overall better values in elastic moduli than gelatin compared to fresh porcine brain in another study [79]. Further research is still needed to establish a suitable and well comparable brain simulant.

#### Bone simulants

Except for torso shot simulations, where bone structures need not be included, and for shots merely focusing on the analysis of, e.g., temporal cavity characterization or gelatin behavior, a solid material bone simulant should be implemented in ballistic model setup. Especially when investigating shots to the head as the most important target area in (molecular) ballistic research, a rigidly cased model is needed, as full casing will strongly influence energy distribution and temporal cavity formation and consequently forward and backspatter behavior [86, 87]. Earlier ballistic head models in molecular ballistics employed acrylic spheres [17] or polyethylene bottles [20, 21, 51] to simulate the skull bone or included no bone simulant at all [52]. Acryl or polyethylene while being sturdy and stable certainly exhibit quite different mechanical properties than human bone. Euteneuer et al. in 2019 presented the first skull model composed of dedicated bone simulant (SYNBONE) in molecular ballistics [37]. SYNBONE bone surrogates had been used before in several ballistic studies and were generally approved of while acknowledging microscopical differences. Zwirner et al. re-enacted cases of suicides by intraoral detonation of firecrackers using SYNBONE skulls as well and could recreate similar lethal fracture patterns [88]. Bolliger et al. used a SYNBONE pelvis and recommended its use [89]. Taylor and Kranioti used SYNBONE spheres for trauma evaluation and concluded that they performed well as crania proxy, yet behaving more brittle than real bone [90]. Smith et al. not only fired several modern and archaic projectile weapons at SYNBONE spheres and plates and found that they behaved similar to bone but also called for caution when examining or testing for details [91]. Bir et al. investigated SYNBONE as well as Sawbone (Vashon Island, USA) hollow bone surrogates for their use in ballistic testing by comparison to femurs of postmortem human specimens, and while SYNBONE performed better than Sawbone, they concluded that both do not act as an ideal bone surrogate [80]. Still in summary, SYNBONE bone simulants currently appear to be the best suited and most versatile artificial bone simulants.

##### Skin simulants

For molecular ballistic studies, skin simulants have not been thoroughly evaluated and compared so far. Still, especially when investigating contact shots and/or head shot simulations, the skin/scalp serves an important role for backspatter generation by enabling the simulation of a subcutaneous gas pocket [43]. When devising a new model, it is advisable to consider which material will best suit the intended purpose. Artificial skin simulants for ballistic testing mainly aim for comparable values in tensile moduli and tensile strength compared to human (or animal) skin, i.e., the force needed for a projectile to stretch, crush, and rupture the skin. For this, a projectile needs to exceed a threshold velocity or limiting velocity beyond which a penetration of the skin will occur [82]. Still, human skin properties and thickness differ inter-individually depending on physiology, age, sex, etc. [92, 93]. Therefore, only a certain range of skin conditions can be represented by any model. Most values applied in studies go back to insights gleaned from cadavers, e.g., as reported by Tausch, Missliwetz, and DiMaio [94–96], and projections on threshold velocities have been calculated accordingly since [97].

Jusilla et al. also relied on these values and tested different materials, concluding that cowhide with semi-finished chrome-tanned upholstery “crust” most closely emulates human skin, while acknowledging the general problem with variation in biological simulants [82]. The skin simulant used in the “skin-skull-brain” model by Thali et al. consists of silicon with artificial fibers and produced results comparable to real cases [71], while Felland-Cheung et al. described dental silicon as an alternative for skin in a study comparing dental materials as simulants to fresh porcine skin [83]. Das et al. evaluated simulant materials for cranial backspatter and while only employing very limited materials, they concluded that lorica leather is a better skin simulant than natural rubber for backspatter testing [84]. In a recent study, Pullen et al. evaluated Roebuck 1518 synthetic chamois (RBK) backed by 10% gelatin for ballistic and forensic use and confirmed this material’s suitability as skin simulant with test using non-deforming projectiles [85]. Other artificial materials devised with comparable properties are emerging, like the Artificial Skin Model (ASM) by Nachmann and Franklin, but these are in need for tests in ballistic experiments first [98].

#### Triple contrast

To aid investigations and sample collection after gunshot incidents, it is essential to know about the mechanisms influencing forward or backspatter generation and where the traces are to be found. After successfully having tested paint (for visual contrast) and radiological contrast material in ballistic models [99–101], Schyma et al. devised and evaluated a multicomponent mixture for trace analysis dedicated for molecular ballistics to facilitate experimental backspatter analysis, termed triple-contrast method [51]. It comprises blood as a biological source for molecular ballistic analysis, acrylic paint for visual inspection of the gun, barrel, and wound channel, as well as a barium sulfate containing radiological contrast agent for CT analysis of the gelatin after shooting. The unique advantage of triple contrast is that it enables the combination of several analytical aspects from the same shot event. Not only is this efficient and cost saving, it also allows for the integration of and to investigate possible correlations between different insight categories: the pattern and distribution of backspatter traces but also wound channel morphology with cracks and fissures within the model is visualized via the acrylic paint and wound channel analysis applying, e.g., the polygon method [99, 102] can be performed. However, before the model is cut into slices, the radiocontrast agent component enables 3D capturing of the wound channel using CT. The wound channel characteristics as assessed visually and by radioimaging can then be related to backspatter trace patterns outside and inside the gun as well as to nucleic acid yields extracted from the blood component in the backspatter by molecular ballistic analysis. Various studies employed the triple-contrast mix in different concentrations, and in no case has PCR inhibition attributable to the mixture components been reported [20, 37, 52]. If the visual contrast aspect is not needed, acrylic paint can be omitted from the mixture, because, depending on brand and physical characteristics like viscosity and density, it can adhere tightly to firearm alloys requiring time and effort to remove, thereby complicating and prolonging the cleaning process (see “Cleaning guns”) at a shooting session. Also, the use of a “double-contrast” variation, with only blood and contrast agent in a 1:1 mixture was recently described also with no observable inhibition [38].

#### Cleaning guns

An important aspect in molecular ballistic experimentation is an effective cleaning procedure to remove all remaining and especially biological traces before performing a subsequent shot with the same gun. Also, cleaning effectiveness should always be assessed by taking negative samples from the freshly cleaned gun. Methods and materials employed in the cleaning procedure should be suitable to be applied at the shooting site thus are neither excessively time consuming nor too complicated. Care should be taken to avoid that the procedure damages or destroys the gun’s alloy, coating, or other parts. This should be checked in advance, and we advise to obtain information about the materials and manufacturing process of the weapons to be used and to test whether, for instance, a cleaning agent intended to remove traces of nucleic acids may fret at the gun’s surfaces. Consequently, studies on molecular ballistics describe mechanical cleaning as the method of choice, employing pieces of woolen felt for cleaning the inside of the barrel, and different chemical substances, like Roti® Nucleic Acid-Free (Carl Roth GmbH, Germany) [21, 38], DNAExitusPlus (AppliChem GmbH, Germany) [52], purpose-made ballistic oil “Ballistol” (F.W. Klever GmbH, Aham, Germany) [17], or 10% bleach (DanKlorix, Colgate-Palmolive, Germany) [37]. The latter, however, is not to be recommended as it may dissolve the coating of the gun and cause corrosion, which may even endanger the shooter when reusing the weapon. Cleaning a gun in an ultrasonic bath filled with an appropriate DNA cleaning agent may be an elaborate yet expensive alternative; however, the weapon needs to be dried and oiled thoroughly afterwards.

## Future research and applications

### Application to cold cases

“Cold cases” are unsolved criminal investigations which remain open pending the discovery of new evidence. It seems promising to apply molecular ballistics to the revision of such cases with gun-related injuries or deaths if the firearm in question had not been probed from the inside during the original investigation, which will regularly be the case. Backspatter on inner surfaces may then still persist, can be recovered and analyzed after years of storage, and can remain analyzable even after standard ballistic test shootings have been performed with the gun [18]. For instance, Schyma et al. generated a full DNA profile from backspatter recovered from a cold case gun that had been in police custody for about 10 years (case no. 15 in [18]). Thus, traces of backspatter containing the DNA (and/or RNA) of one or more of the victims of a particular gun may result in new and unexpected investigative leads in cold cases even after many years.

### Massive parallel sequencing

Massive parallel sequencing (MPS), also often referred to as “next-generation sequencing” (NGS), is an emerging key technology in genetics and genomics [103] enabling the simultaneous (parallel) sequencing of millions of nucleic acid fragments which allows for whole genomes to be sequenced in a single day for less than $1000 [104]. MPS bears outstanding potential also for forensic molecular biology [105] and has been introduced to forensic research in 2010 [106]. Employing MPS in routine forensic DNA analysis of trace material enhances success rates with minute amounts of and/or degraded material and considerably increases allelic discrimination as equal length alleles of different contributors can be differentiated via minuscule differences in sequence. In addition, more information can be yielded from the same item of evidence because different types of nucleic acids can be extracted and sequenced in parallel, including genomic DNA, mtDNA, mRNA, and miRNA, hence combining individualization and contextualization of trace material [107] and even facilitating assignment of body fluids to donors in mixed blood stains [108]. Also, there are forthcoming promising results from current forensic RNA research and technological advances including MPS which will eventually allow forensic RNA analysis of biological trace material to determine the time elapsed as well as the time of day (“molecular alibi”) that a crime was committed [109–113]. Integrating such progressive approaches with molecular ballistic analyses will yield even more contextual information from biological material recovered from gun-related crimes that can support the reconstruction of critical temporal details of the shooting event. These possibilities clearly encourage further research for and recommend applying MPS in molecular ballistic analyses where small amounts of challenged material are frequently encountered. In fact, Hanson and Ballantyne already demonstrated the potential of RNA-based OTI via MPS to support the investigation of gun-shot and other traumatic injuries [114].

### Blank guns

**Fig. 6:**
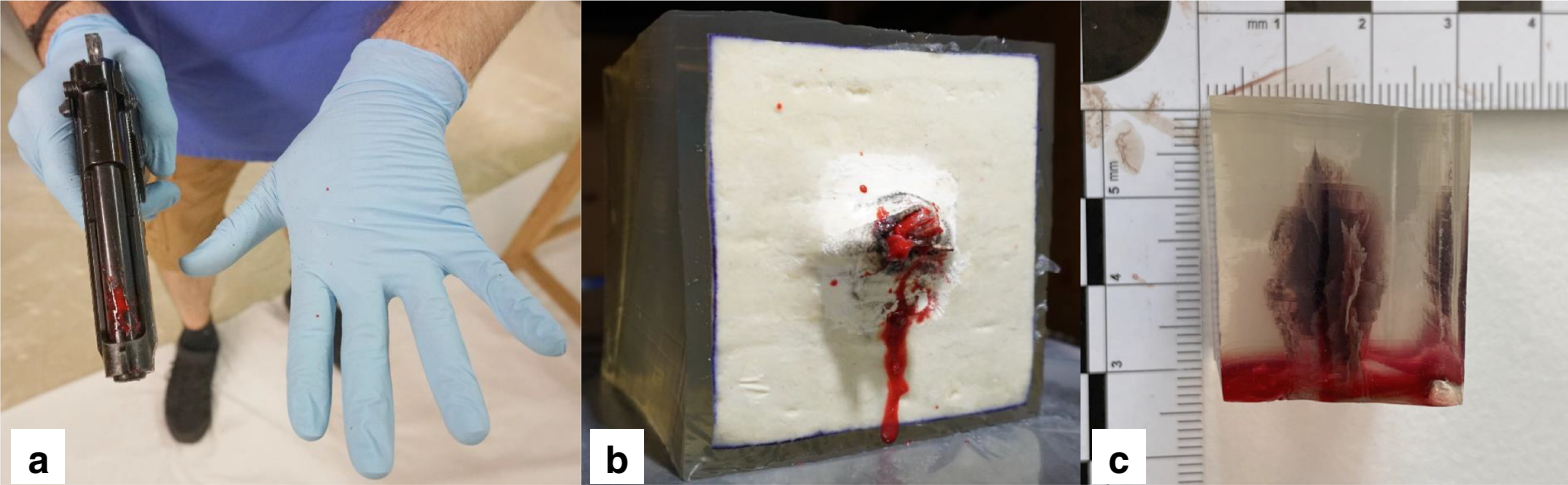
Photos taken during experimental shooting session of Euteneuer et al. (manuscript in review). **a** Ekol Firat blank pistol and shooter’s hands with backspatter traces after contact shot with Skullfire 9 mm P.A.K. ammunition (Pobjeda Technology, Bosnia and Herzegovina) to the gelatine cube at (**b**). **b** Gelatin cube with chamois leather as skin simulant and doped with “double-contrast” mix in plastic bag after shot with blank gun at (**a**). **c** Cut-out wound channel in gelatin cube created by Ekol Firat shot with Özkursan 9 mm P.A. ammunition

Blank guns, or blank firing/cartridge guns, are underrepresented as a research subject and underrated as a threat to human beings. There are numerous reports on cases and incidents of injury and death involving these weapons suggesting a substantial hazard potential, which are presented comprehensively in a recent study on the analysis of backspatter generated by blank gun contact shots at ballistic models by Euteneuer et al. [81]. They demonstrated that backspatter will reproducibly be created by blank guns of different types and calibers, loaded with different types of ammunitions that are fired at ballistic models. The gas jets of those shots created pronounced wound channels in the gelatin block models used in their study (Fig. 6), which corresponds to case reports on severe and lethal wounds in human bodies, thus again demonstrating a considerable harming potential of blank gunshots. This proof-of-concept study therefore opens the field for and indicates a need of further molecular ballistic research on firearms with no projectiles.
